# Supplementary material for: A comprehensive molecular profiling approach reveals metabolic alterations that steer bone tissue regeneration
Source: Commun Biol. 2023 Mar 27;6:327. doi: 10.1038/s42003-023-04652-1 (PMC10042875; doi:10.1038/s42003-023-04652-1)
Supplement: Supplementary file 2 — Supplementary Information [file 42003_2023_4652_MOESM2_ESM.pdf]

Supplementary Information to:

A comprehensive molecular profiling approach reveals metabolic alterations that steer bone tissue regeneration

Julia Löffler[1,2,3], Anne Noon[1,2], Agnes Ellinghaus[1,2], Anke Dienelt[1,2], Stefan Kempa[3]†* and Georg N. Duda[1,2]† *

[1] Julius Wolff Institute (JWI), Berlin Institute of Health at Charité – Universitätsmedizin Berlin, 13353 Berlin, Germany

[2] BIH Center for Regenerative Therapies (BCRT), Berlin Institute of Health at Charité – Universitätsmedizin Berlin, 13353 Berlin, Germany

[3] Berlin Institute for Medical Systems Biology, Max-Delbrück-Center for Molecular Medicine, 10115, Berlin, Germany

† These authors contributed equally to this work as senior author

Running title: Metabolic alterations steer bone regeneration

*Correspondence:

Prof. Dr.-Ing. Georg N. Duda, Julius Wolff Institute and Berlin Institute of Health Center for Regenerative Therapies at Berlin Institute of Health and Charité – Universitätsmedizin Berlin, Augustenburger Platz 1, D-13353 Berlin, Germany, e-mail: georg.duda@charite.de, telephone: +49 30 450559079, fax: +49 30 450 559969

Supplement Methods

Experimental design – animal model

Surgeries were performed on two groups: (1) 3 month old female Sprague-Dawley rats and (2) 12 months old female ex-breeder Sprague-Dawley rats that have had a minimum of three litters (weight 400±60 g), purchased at Charles River. Aged animals were purchased aged 7-8 months, with the last litter was born approximately 4-6 weeks before delivery to our animal housing. Since the companies do not keep the animals after exclusion from breeding, animals were kept for 4-5 months in our in-house facilities before surgical procedure. Previously published studies by Preininger et al., Strube et al. and Löffler et al. confirm these animals as a model for biologically compromised fracture healing, resulting in the formation of a non-union without further treatment during the investigation period [1-5].

Animals received a 2mm osteotomy at the left femur under general anesthesia of 0.3 mg/kg Medetomidin DomitorH and 60 mg/kg Ketamin administered i.p.. Additionally, 45 mg/kg Clindamycin was administered by s.i. and eyes were prevented from drying out by application of eye balm. For analgesia prior to the surgery the animals received 20 mg/kg Tramadol. A longitudinal skin incision was made over the left femur and the bone exposed by blunt fascia dissections. An in-house developed unilateral external fixator was mounted to stabilize the bone, made of stainless steel and titanium as published previously. [3] To ensure the highest possible standardization for placement of the four wire holes, a drilling template was used for every surgery. After incision of the titanium wires, the external fixator bar was placed on the wires and a standardized 2mm gap was sawn into the femoral bone. To ensure gap size reproducibility a sawing template was used at all times. Muscle fascia and skin were closed using absorbable and non-absorbable sutures, respectively. Tramadol as post-surgery analgesia was administered for three days through the drinking water (25 ml/l). Animals were checked

daily for overall health status and fixator positioning. Bone healing was monitored over a period of 2 weeks. Animals were sacrificed and femurs were sampled for histological assessment, gene expression, protein, and metabolite levels at day 3, 7 and 14 after osteotomy. Animals were assigned to the different groups randomly.

For samples not only the fracture tissue but also adjacent tissue, located 1 mm distal and proximal to the fracture hematoma was samples. This was done aiming to include cells and signals from the bone marrow that are attracted to the fracture site. This is especially relevant in later time points of the healing cascade, where relevant players migrate from the bone marrow cavity into the fracture gaps. In order to obtain tissue samples that can be compared in the best possible/standardized way this approach was chosen.

Supplementary Figures

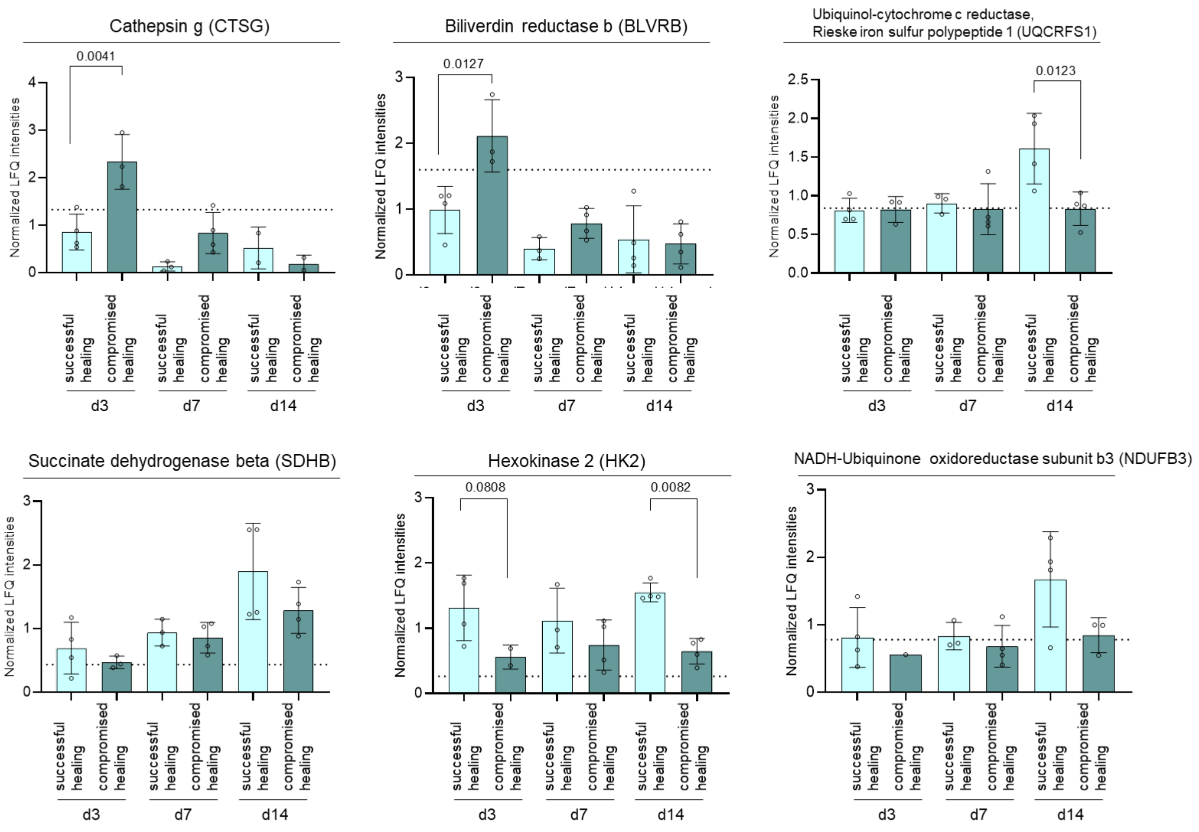

Supplementary figure 1. Exemplary proteins and their abundance for the identified and regulated protein cluster in successful vs. compromised healing. Cathepsin G and biliverdin reductase are representative for higher abundance of pro-inflammatory proteins in compromised healing, while ubiquinol-cytochrome c reductase, Rieske iron sulfur polypeptide 1 and NADH-ubiquinone oxidoreductase subunit 3 show exemplary levels for oxidative metabolism. Succinate dehydrogenase beta and hexokinase 2 levels are representative higher for central carbon metabolism protein abundance in successful healing samples. The dotted line represents respective protein abundance in unfractured, contralateral femoral bone. Shown are mean±standard deviation, normalized label-free-quantification (LFQ) intensities, n=3-5 individual biological replicates per group and time point, One-way ANOVA.

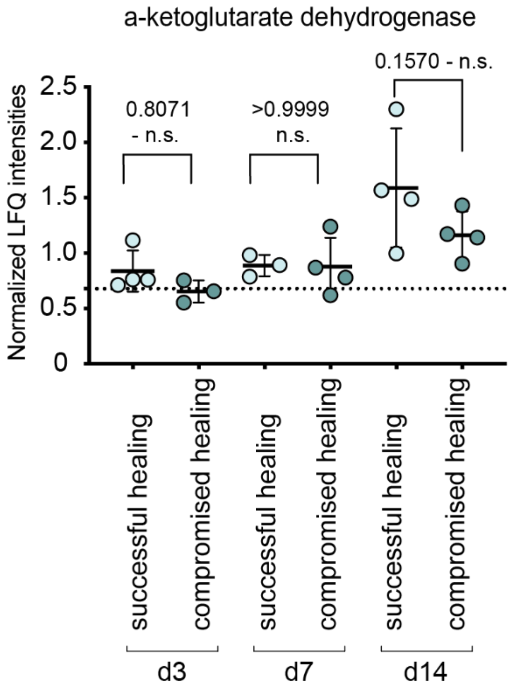

Supplementary figure 2. OGDH protein levels between successful and compromised healing. OGDH protein levels showed an increase at d14 after osteotomy in both healing groups (successful and compromised). The dotted line represents OGDH levels in unfractured, contralateral femoral bone. Shown are mean±standard deviation, normalized label-free-quantification (LFQ) intensities, n=3-5 individual biological replicate, per group and time point, One-way ANOVA.

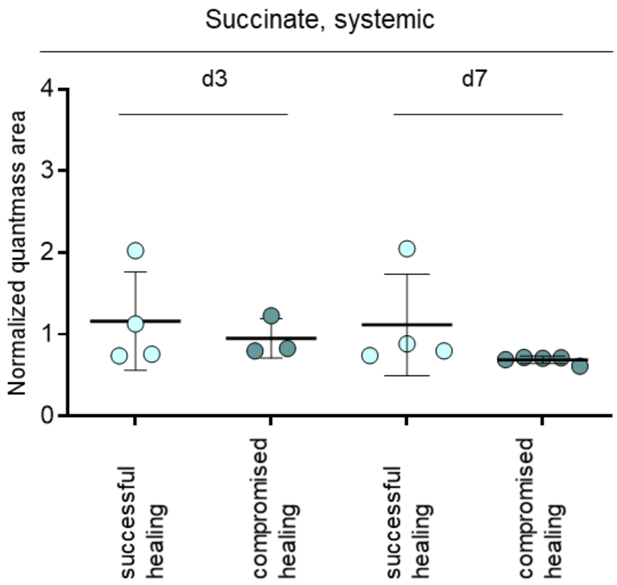

Supplementary figure 3. Systemic levels of succinate show now alterations between healing time points or groups.

Analysis of systemic succinate levels, from intracardiac blood collection showed no significant difference between compromised and successful healing at fracture healing onset (day 3, day 7) nor significant level alterations between day 3 and day 7 within healing groups. Mean±standard deviation, n=3-5 individual biological replicates per group and time point, t-test.

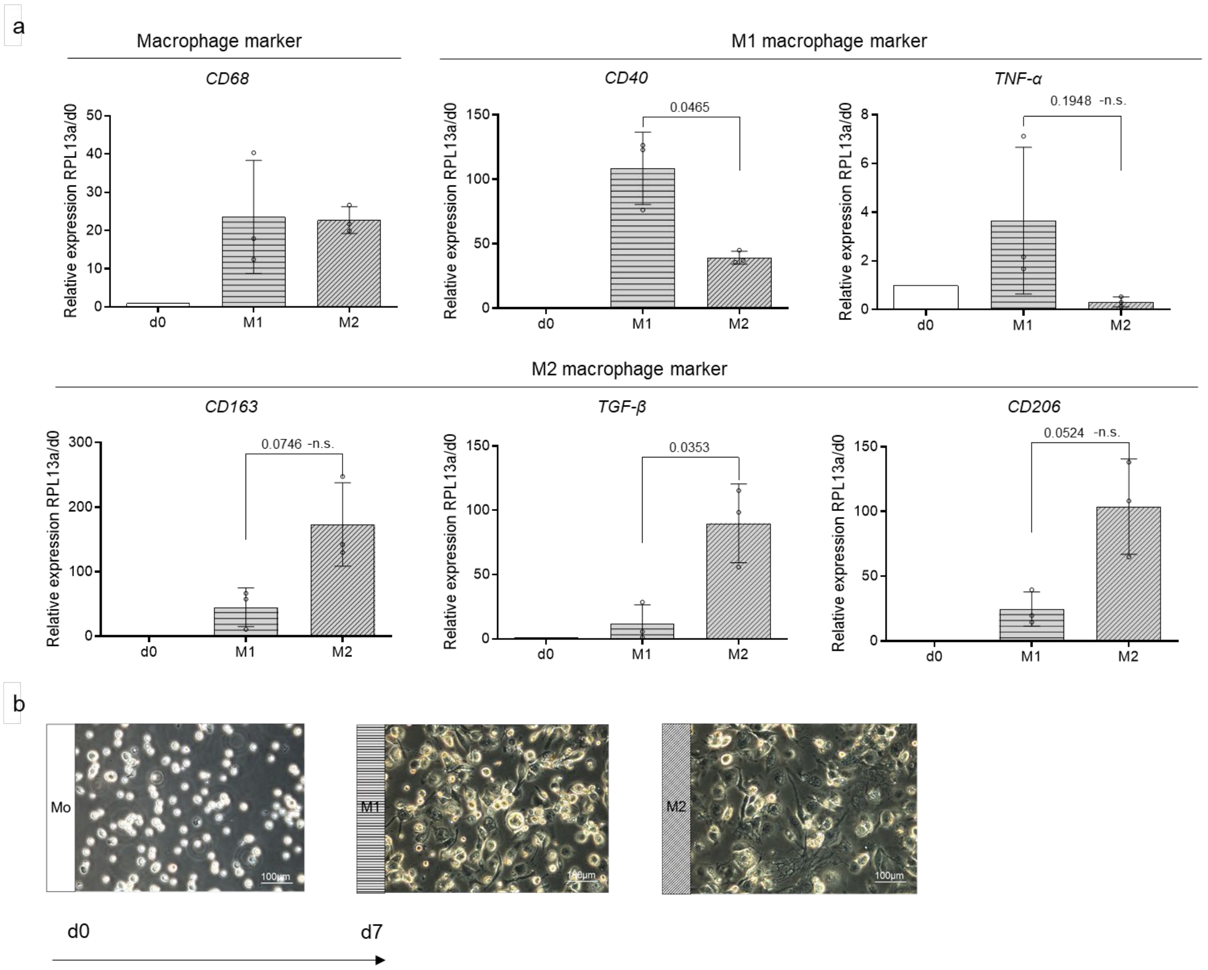

Supplementary figure 4. THP-1 human monocytic cell line differentiation into M1 and M2 macrophages. (a) The general marker for macrophages *CD68* is upregulated upon differentiation from monocytes to macrophages in both M1 and M2 polarized macrophages. *CD40* and *TNF-α* marker for M1 macrophage polarization are upregulated in M1 polarized macrophages, while *TGF-β* and *CD206* – markers for the M2 subtype are upregulated in M2 polarized macrophage cultures. Relative expression to RLP13a (housekeeping gene and day 0). 3-4 individual experimental replicates, mean±standard deviation, One-way ANOVA. (b) Microscopic images of THP-1 monocytes (suspension cells) before differentiation (20x magnification). Microscopic images after 7 days of differentiation and polarization, M1 polarized cultures (+LPS, +INFγ) and M2 polarized cultures (+IL-4, +IL-13) (20x magnification).

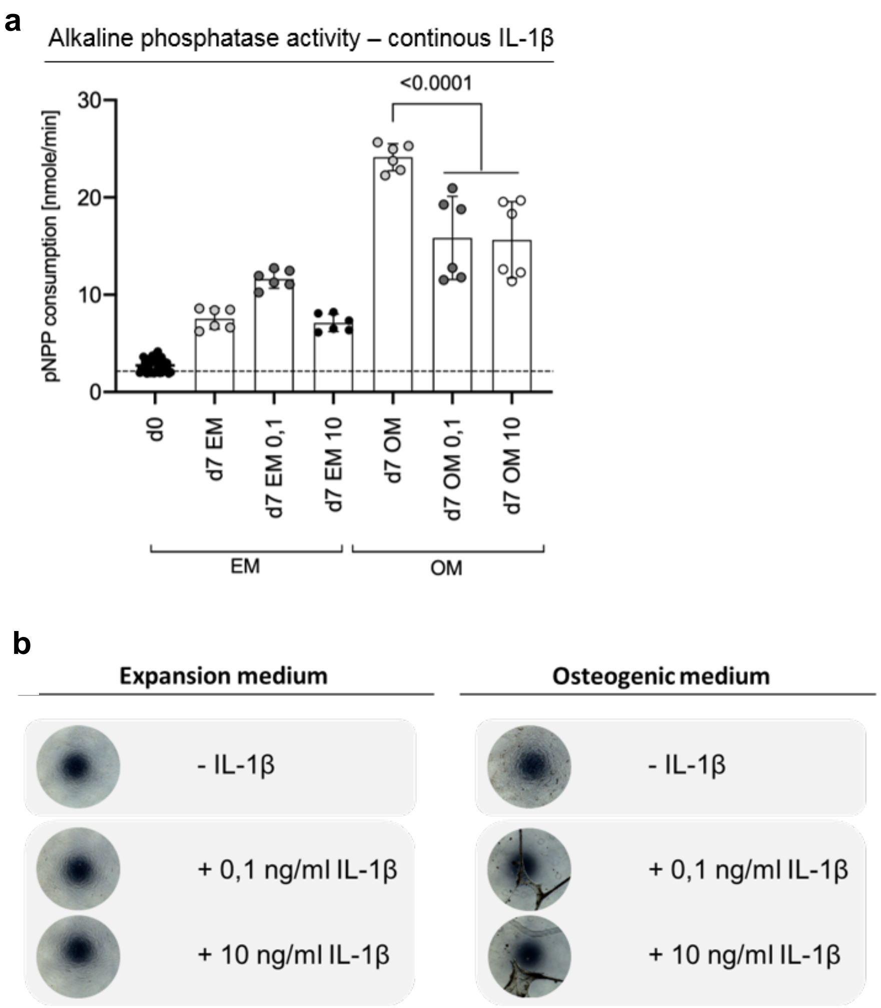

Supplementary figure 5. Constant exposure of IL-1β strongly diminished the osteogenic differentiation capacity of primary human MSCs in vitro. (a) Addition of 0.1ng/ml and 10ng/ml IL-1β to osteogenic cultures, did not enhance osteogenic differentiation, as the early marker for osteogenic differentiation, alkaline phosphatase activity, was significantly reduced after 7 days of culture, pNPP - 4-nitrophenylphosphate. 6 individual experimental replicates, mean±standard deviation, One-way ANOVA. (b) The cells in osteogenic conditions showed a strong contraction and loss of cell layer integrity when IL-1β was added to the culture, making further analysis impossible, as shown in the microscopic images.

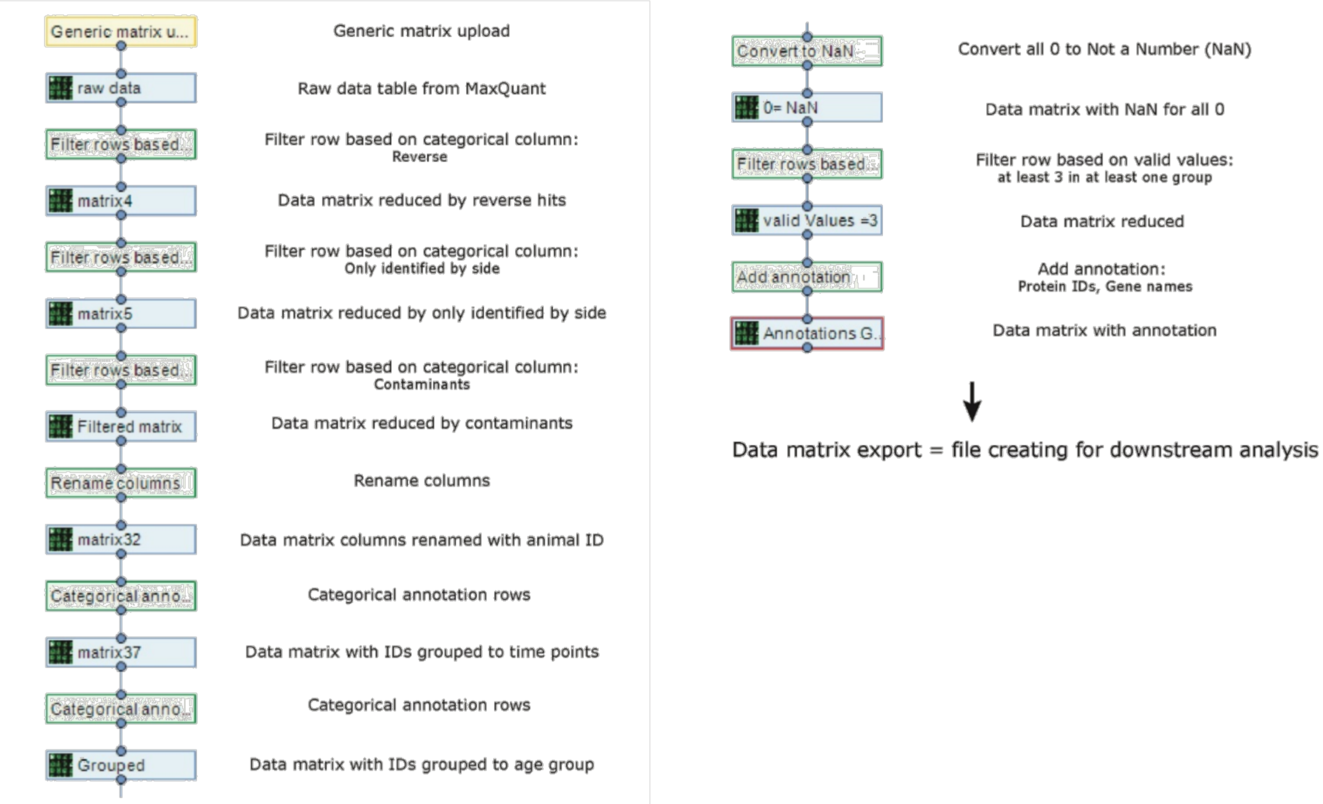

Supplementary figure 6. Schematic workflow of the processing of the data matrix from MaxQuant using the Perseus software. Data matrix from MaxQuant is uploaded into Perseus, contaminants and cleavage errors were excluded. Numerical IDs were replaced with animal IDs, respective time point and grouped accordingly. All data points were no peptides were measured (0) were replaced to NaN and matrix was filtered and reduced to proteins identified at least 3 times per group (age). By upload of the uniprot data base: rattus norvegius protein IDs and gene names were added to the data. The data matrix was then exported for further analysis.

Supplementary Tables

Supplementary Table 1. Animal treatment during surgical procedure.

Substance	Concentration	Mode administered	Aim	Time point	Distributor
Medetomidin DomitorH	0.3mg/kg	Intraperitoneal injection	Anesthetic	Before surgery	OrionPharma, Germany
Ketamin	60mg/kg	Intraperitoneal injection	Anesthetic	Before surgery	Actavis, Ireland
Tramadol	20mg/kg	Intraperitoneal injection	Analgesia	Before surgery	Grünenthal, Germany
Clindamycin	45mg/kg	Subcutaneous injection	Antibiotic	Before surgery	Ratiopharm, Germany
Bepanthen® eye balm	-	Applied on eyes	Prevention of corneal drying out	Before surgery	Bayer Vital GmbH, Germany
Antisedan	1.5 mg/kg	Intraperitoneal injection	Anti-anesthetic	Post-surgery	Pfizer, Germany
Tramadol	25ml/l	Drinking water	Analgesia	3 days post-surgery	Grünenthal, Germany

Supplementary Table 2. Primer pairs and sequences.

Species	Gene	Gene Name	Forward 5´-3´	Reverse 5‘-3‘
human	*CD40*	Cluster of differentiation 40	ggtctcacctcgctatggt	cagtgggtggttctggatg
	CD68	Cluster of differentiation 68	gtccacctcgacctgctct	cactggggcaggacaaact
	CD163	Cluster of differentiation 163	tcagctgatttcagtgctgct	aggctgaactcactgggttataaat
	CD206	Cluster of differentiation 206	gggccaagcttctctggaat	tttatccacagccacgtccc
	TNF-α	Tumor necrosis factor alpha	tccccagggacctctctcta	gagggtttgctacaacatggg
	TGF-β	Transforming growth factor beta	gcgtgctaatggtggaaacc	gcttctcggagctctgatgt
	IL-1β	Interleukin -1 beta	gcgtgctaatggtggaaacc	gcttctcggagctctgatgt
	RPL13A	Ribosomal Protein 13a	cctggaggagaagaggaaagaga	ttgaggacctctgtgtatttgtcaa
rat	*Col1a2*	Collagen type I alpha chain 2	ggagagagtgccaactccag	ccaccccagggataaaaact
	Spp1	Osteopontin	gaggagaaggcgcattacag	atggctttcattggagttgc
	Tbp	Tata-box binding protein	ggaccagaacaacagccttc	ccgtaaggcatcattggact

Supplementary References

1 Preininger, B. *et al.* An Experimental Setup to Evaluate Innovative Therapy Options for the Enhancement of Bone Healing Using Bmp as a Benchmark - a Pilot Study. *Eur Cells Mater* **23**, 262-272, doi:Doi 10.22203/Ecm.V023a20 (2012).

2 Strube, P. *et al.* Sex-specific compromised bone healing in female rats might be associated with a decrease in mesenchymal stem cell quantity. *Bone* **45**, 1065-1072, doi:https://doi.org/10.1016/j.bone.2009.08.005 (2009).

3 Strube, P. *et al.* A new device to control mechanical environment in bone defect healing in rats. *J Biomech* **41**, 2696-2702, doi:10.1016/j.jbiomech.2008.06.009 (2008).

4 Löffler, J. *et al.* Compromised Bone Healing in Aged Rats Is Associated With Impaired M2 Macrophage Function. *Frontiers in Immunology* **10**, doi:10.3389/fimmu.2019.02443 (2019).

5 Mehta, M., Duda, G. N., Perka, C. & Strube, P. Influence of gender and fixation stability on bone defect healing in middle-aged rats: a pilot study. *Clinical orthopaedics and related research* **469**, 3102-3110, doi:10.1007/s11999-011-1914-y (2011).
