# Supplementary material for: A comprehensive molecular profiling approach reveals metabolic alterations that steer bone tissue regeneration
Source: Commun Biol. 2023 Mar 27;6:327. doi: 10.1038/s42003-023-04652-1 (PMC10042875; doi:10.1038/s42003-023-04652-1)
Supplement: Supplementary file 3 — Description of Additional Supplementary Files [file 42003_2023_4652_MOESM3_ESM.pdf]

Description of Additional Supplementary Files

Supplementary Data 1

The source data behind graphs in figure 1 in main manuscript

Supplementary Data 2

The source data behind graphs in figure 2 in main manuscript

Supplementary Data 3

The source data behind graphs in figure 3 in main manuscript

Supplementary Data 4

The source data behind graphs in figure 4 in main manuscript

Supplementary Data 5

The source data behind graphs in figure 5 in main manuscript

Supplementary Data 6

The source data behind graphs in figure 6 in main manuscript

Supplementary Data 7

The source data behind graphs in figure 7 in main manuscript

Supplementary Data 8

The source data behind graphs in figure 8 in main manuscript

Supplementary Data 9

The source data behind graphs in figure 9 in main manuscript

Supplementary Data 10

The source data behind graphs in figure 10 in main manuscript

Supplementary Data 11

The source data behind graphs in Supplementary Figure 1 in supplementary information

Supplementary Data 12

The source data behind graphs in Supplementary Figure 2 in supplementary information

Supplementary Data 13

The source data behind graphs in Supplementary Figure 3 in supplementary information

Supplementary Data 14

The source data behind graphs in Supplementary Figure 4 in supplementary information

Supplementary Data 15

The source data behind graphs in Supplementary Figure 5 in supplementary information

Supplementary Data 16

Supplementary Data 16 referenced in the main manuscript

Supplementary Data 17

Supplementary Data 17 referenced in the main manuscript
