# Supplementary material for: A comprehensive molecular profiling approach reveals metabolic alterations that steer bone tissue regeneration
Source: Commun Biol. 2023 Mar 27;6:327. doi: 10.1038/s42003-023-04652-1 (PMC10042875; doi:10.1038/s42003-023-04652-1)
Supplement: Supplementary file 21 — nr-reporting-summary_COMMSBIO-22-0835A_final_submission [file 42003_2023_4652_MOESM21_ESM.pdf]

nature portfolio

Corresponding author(s): Prof. Georg N. Duda | georg.duda@charite.de

Last updated by author(s): Feb 7, 2023

Reporting Summary

Nature Portfolio wishes to improve the reproducibility of the work that we publish. This form provides structure for consistency and transparency in reporting. For further information on Nature Portfolio policies, see our Editorial Policies and the Editorial Policy Checklist.

Statistics

For all statistical analyses, confirm that the following items are present in the figure legend, table legend, main text, or Methods section.

n/a	Confirmed	
☐	☒	The exact sample size (*n*) for each experimental group/condition, given as a discrete number and unit of measurement
☐	☒	A statement on whether measurements were taken from distinct samples or whether the same sample was measured repeatedly
☐	☒	The statistical test(s) used AND whether they are one- or two-sided<br>*Only common tests should be described solely by name; describe more complex techniques in the Methods section.*
☐	☒	A description of all covariates tested
☐	☒	A description of any assumptions or corrections, such as tests of normality and adjustment for multiple comparisons
☐	☒	A full description of the statistical parameters including central tendency (e.g. means) or other basic estimates (e.g. regression coefficient) AND variation (e.g. standard deviation) or associated estimates of uncertainty (e.g. confidence intervals)
☐	☒	For null hypothesis testing, the test statistic (e.g. *F*, *t*, *r*) with confidence intervals, effect sizes, degrees of freedom and *P* value noted<br>*Give P values as exact values whenever suitable.*
☒	☐	For Bayesian analysis, information on the choice of priors and Markov chain Monte Carlo settings
☒	☐	For hierarchical and complex designs, identification of the appropriate level for tests and full reporting of outcomes
☒	☐	Estimates of effect sizes (e.g. Cohen's *d*, Pearson's *r*), indicating how they were calculated

Our web collection on statistics for biologists contains articles on many of the points above.

Software and code

Policy information about availability of computer code

Data collection	Imaging AxioVision 4.4 LE Software (Carl Zeiss, Germany)<br>ChromaTOF® (Leco, United States)<br>MaxQuant & Andromeda (Cox J, Mann M. MaxQuant enables high peptide identification rates, individualized p.p.b.-range mass accuracies and proteome-wide protein quantification. Nature Biotechnology. 2008;26:1367.<br>Cox J, Hein MY, Luber CA, Paron I, Nagaraj N, Mann M. Accurate Proteome-wide Label-free Quantification by Delayed Normalization and Maximal Peptide Ratio Extraction, Termed MaxLFQ. Molecular & Cellular Proteomics. 2014;13(9):2513-26)
Data analysis	Graph Pad Prism 9.5.0 (United States)<br>Perseus (Tyanova S, Temu T, Sinitcyn P, Carlson A, Hein MY, Geiger T, et al. The Perseus computational platform for comprehensive analysis of (prote)omics data. Nature Methods. 2016;13:731)<br>MAUI VIA (Kuich PHJL, Hoffmann N, Kempa S. Maui-VIA: A User-Friendly Software for Visual Identification, Alignment, Correction, and Quantification of Gas Chromatography–Mass Spectrometry Data. Frontiers in Bioengineering and Biotechnology. 2014;2:84)<br>ImageJ (Version 1.44p, United States)<br>TScratch (Geback T, Schulz MMP, Koumoutsakos P, Detmar M. TScratch: a novel and simple software tool for automated analysis of monolayer wound healing assays. Biotechniques. 2009;46(4):265-+.)

For manuscripts utilizing custom algorithms or software that are central to the research but not yet described in published literature, software must be made available to editors and reviewers. We strongly encourage code deposition in a community repository (e.g. GitHub). See the Nature Portfolio guidelines for submitting code & software for further information.

Data

Policy information about availability of data

All manuscripts must include a data availability statement. This statement should provide the following information, where applicable:

- Accession codes, unique identifiers, or web links for publicly available datasets
- A description of any restrictions on data availability
- For clinical datasets or third party data, please ensure that the statement adheres to our policy

The mass spectrometry proteomic datasets generated and analyzed have been deposited to the ProteomeXchange Consortium (http://proteomecentral.proteomexchange.org) via the PRIDE (70) partner repository with the dataset identifier PXD020085. All other source data used for figure generation are available in Excel format according to the journals policy.

Human research participants

Policy information about studies involving human research participants and Sex and Gender in Research.

Reporting on sex and gender	Sex and gender have not been differentiated for the use of primary cells (MSCs) in this study.
Population characteristics	Cells (MSCs) from human biospecimen were used for this study. All biospecimen were collected only after informed and written consent and according to the EA099/10 ethics approval.
Recruitment	For this study no extra patients have been recruited. Used cells have been rountinely collected according to the EA009/10 ethics approval that allows the collection of biospecimen during hip or joint replacement surgeries and after written consent. Cells were then isolated, characterized, expanded, passaged and stored according to previously published and referenced protocols.
Ethics oversight	Ethics approval EA099/10, LaGeSo

Note that full information on the approval of the study protocol must also be provided in the manuscript.

Field-specific reporting

Please select the one below that is the best fit for your research. If you are not sure, read the appropriate sections before making your selection.

☒ Life sciences ☐ Behavioural & social sciences ☐ Ecological, evolutionary & environmental sciences

For a reference copy of the document with all sections, see nature.com/documents/nr-reporting-summary-flat.pdf

Life sciences study design

All studies must disclose on these points even when the disclosure is negative.

Sample size	For all cell culture experiments samples were determined as a multiple measurements/replicates (minimum of triplicate measurements) during one experimental set-up (technical replicate). Technical replicates (with a minimum of 3 replicate measurements) were performed three times (minimum) independently.
Data exclusions	Number of biological replicates in the experimental groups may vary due to termination rules for animal experiments or technical failure during measurements (e.g. sample quality low during MS-measurement, as determined by internal standards). In this case samples were excluded from further processing and subsequently analysis.
Replication	Reproducibility of experiments was confirmed by replication. Number of technical and biological replicates are indicated in figure captions, as are the applied statistical methods.
Randomization	Sample/animal allocation were randomized, as was metabolomic and proteomic sample meassurement sequence.
Blinding	Where possible blinded analysis of data was performed (e.g. histomorphometric analysis, tube formation analysis and migration assasy). Peak annotation of raw data for metabolomics and proteomics was also done blinded, since samples were randomized and pseudomized before measurement. De-blinding was only performed, when experimental group specific statistical testing was performed. Due to different age and healing timepoints, collection of rat hematoma specimen was not blinded, but since blinded data approaches were used, where applicable we believe this has no effect on data quality.

Reporting for specific materials, systems and methods

We require information from authors about some types of materials, experimental systems and methods used in many studies. Here, indicate whether each material, system or method listed is relevant to your study. If you are not sure if a list item applies to your research, read the appropriate section before selecting a response.

Materials & experimental systems

n/a	Involved in the study
☒	☐ Antibodies
☐	☒ Eukaryotic cell lines
☒	☐ Palaeontology and archaeology
☐	☒ Animals and other organisms
☒	☐ Clinical data
☒	☐ Dual use research of concern

Methods

n/a	Involved in the study
☒	☐ ChIP-seq
☒	☐ Flow cytometry
☒	☐ MRI-based neuroimaging

Eukaryotic cell lines

Policy information about cell lines and Sex and Gender in Research

Cell line source(s)	Commercial cell lines used: THP-1 cell line (human), HUVECs (human)<br>Primary cells used: human MSCs from individual donors (anonymized use - no sex differentiation)
Authentication	Commercial cell lines - THP-1: ECACC listed and purchased from Sigma Aldrich/Merck 88081201-1VL, HUVECs purchased from Lonza<br>Commercial cell lines were provided with CoA/COO upon shipping.<br>Primary MSCs cell lines where checked for prerequistief of the International Society for Cellular Therapy, as stated in the methods section of the manuscript.
Mycoplasma contamination	Cell lines (and all cell cultures) tested negative for mycoplasma contamination at first thaw and were regularly tested for mycoplasma contamination during culture. No contamination was detected at any time.
Commonly misidentified lines<br>(See ICLAC register)	N/A

Animals and other research organisms

Policy information about studies involving animals; ARRIVE guidelines recommended for reporting animal research, and Sex and Gender in Research

Laboratory animals	This manuscript used 3- and 12-months old female ex-breeder Sprague Dawley rats purchased from Charles River WIGA Deutschland GmbH.
Wild animals	This study did not involve wild animals.
Reporting on sex	The here used model focused on female animals only, as they provide a preclinical model of biologically impaired healing without further intervention needed. Detailed information on the here used model and all applicable references are included in the methods section of the manuscript.<br>Follow-up experiments cell culture experiments do not differntiate between sex.
Field-collected samples	This study did not involve field-collected samples.
Ethics oversight	All experiments were in compliance with ARRIVE guidelines, the National Institutes of Health Guide for the Care and Use of Laboratory Animals, and the National Animal Welfare Guidelines. Animal experiments were approved by the local legal representative (Institutional Animal Care and Use Committees, LaGeSo, G0120/14, G0172/15).

Note that full information on the approval of the study protocol must also be provided in the manuscript.
